# The Role of EGFR in Influenza Pathogenicity: Multiple Network-Based Approaches to Identify a Key Regulator of Non-lethal Infections

**DOI:** 10.3389/fcell.2019.00200

**Published:** 2019-09-20

**Authors:** Hugh D. Mitchell, Amie J. Eisfeld, Kelly G. Stratton, Natalie C. Heller, Lisa M. Bramer, Ji Wen, Jason E. McDermott, Lisa E. Gralinski, Amy C. Sims, Mai Q. Le, Ralph S. Baric, Yoshihiro Kawaoka, Katrina M. Waters

**Affiliations:** ^1^Pacific Northwest National Laboratory, Richland, WA, United States; ^2^Department of Pathobiological Sciences, University of Wisconsin–Madison, Madison, WI, United States; ^3^Department of Microbiology and Epidemiology, The University of North Carolina at Chapel Hill, Chapel Hill, NC, United States; ^4^National Institute of Hygiene and Epidemiology, Hanoi, Vietnam; ^5^Division of Virology, Department of Microbiology and Immunology, Institute of Medical Sciences, The University of Tokyo, Tokyo, Japan; ^6^International Research Center for Infectious Diseases, Institute of Medical Sciences, The University of Tokyo, Tokyo, Japan

**Keywords:** systems biology, network topology, influenza, SARS-CoV, data integration

## Abstract

Despite high sequence similarity between pandemic and seasonal influenza viruses, there is extreme variation in host pathogenicity from one viral strain to the next. Identifying the underlying mechanisms of variability in pathogenicity is a critical task for understanding influenza virus infection and effective management of highly pathogenic influenza virus disease. We applied a network-based modeling approach to identify critical functions related to influenza virus pathogenicity using large transcriptomic and proteomic datasets from mice infected with six influenza virus strains or mutants. Our analysis revealed two pathogenicity-related gene expression clusters; these results were corroborated by matching proteomics data. We also identified parallel downstream processes that were altered during influenza pathogenesis. We found that network bottlenecks (nodes that bridge different network regions) were highly enriched in pathogenicity-related genes, while network hubs (highly connected network nodes) were significantly depleted in these genes. We confirmed that this trend persisted in a distinct virus: Severe Acute Respiratory Syndrome Coronavirus (SARS). The role of epidermal growth factor receptor (EGFR) in influenza pathogenesis, one of the bottleneck regulators with corroborating signals across transcript and protein expression data, was tested and validated in additional mouse infection experiments. We demonstrate that EGFR is important during influenza infection, but the role it plays changes for lethal versus non-lethal infections. Our results show that by using association networks, bottleneck genes that lack hub characteristics can be used to predict a gene’s involvement in influenza virus pathogenicity. We also demonstrate the utility of employing multiple network approaches for analyzing host response data from viral infections.

## Introduction

Viruses that are newly introduced to the human population have the potential to be highly pathogenic. While the pathogenicity of these new strains tends to wane as adaptation progresses, emerging viruses, such as highly pathogenic avian influenza strains, are an ever-present threat to human health and the global economy because it is difficult to predict when a new pathogenic strain will appear. The 1918 influenza A virus pandemic claimed 20–100 million lives worldwide (Peiris et al., 2004). Multiple influenza pandemics have emerged since. Most recently, human infections of H7N9 influenza, which first emerged in the spring of 2013, have resulted in 1568 infections including 616 deaths (Food and Agriculture Organization [FAO], 2019). Since 2003, H5N1 avian influenza has caused 860 human infections with a mortality rate of 53% (World Health Organization [WHO], 2018). The 2009 H1N1 pandemic caused less severe disease in humans but spread to nearly 200 countries (Michaelis et al., 2009) and may have contributed to the deaths of an estimated 284,000 people (Dawood et al., 2012). The fact that influenza strains vary greatly in pathogenicity underscores the need to understand the underlying host mechanisms that contribute to the severity of infection so that we are better prepared to alleviate the effects of highly pathogenic strains. Despite the potential for pandemic infection with a highly virulent, highly transmissible new strain of influenza, the current understanding of these mechanisms remains limited.

A major advantage of a systems biology approach to pathobiology is the ability to identify novel, key elements of a biological process, such what regulators are involved in critical processes. High-throughput profiling methods (e.g., transcriptomics) provide powerful tools for examining how entire systems respond to different perturbations such as acute disease. Network reconstruction provides the opportunity to utilize all available data and is a critically important tool for representing complex sets of interactions. For biological systems, network analysis has proven useful for analyzing genetic interactions among genes, as well as protein–protein, protein–DNA, and kinase–substrate interactions (Ideker and Krogan, 2012). In addition, network approaches have attempted to identify regulatory associations between genes and proteins by comparing expression patterns across multiple conditions (Faith et al., 2007; McDermott et al., 2009, 2012). These approaches may capture physical interactions but can also identify more subtle, though equally important, regulatory relationships between gene pairs or within gene clusters. Previous work has shown that prioritization of key regulators based on network topology is superior to simple ranking of differentially expressed genes (McDermott et al., 2011). Our group and others have demonstrated that genes occupying certain topological positions in association networks play important regulatory roles in the biological process being studied (Yu et al., 2007; McDermott et al., 2009, 2016; Mitchell et al., 2013). Network hubs are identified by the degree centrality metric, which is the number of edges associated with any given node. Network bottlenecks are identified by the betweenness centrality metric, which is the number of shortest paths between all pairs of nodes that pass through a given node. These are two of the most studied topological features, yet it is unclear from the literature which of these is the most effective predictor of regulatory function for any given network construction approach or biological context. It is also unclear what distinct regulatory roles each has; such information is important to discern as it may be used to identify targets for therapeutic intervention.

Typically, studies that attempt to uncover the underlying mechanisms of pathogenicity simply compare a single high- and low-pathogenicity strain or dose (Hatta et al., 2001; Kobasa et al., 2007; Cilloniz et al., 2009; Safronetz et al., 2011; Tisoncik-Go et al., 2016). While this approach may allow pathogenicity-related host responses to be identified, it can be difficult to distinguish between responses that are truly tied to pathogenicity and those that are strain-specific. For this study, we use network clustering and topology to compare six influenza strains and mutants of varying pathogenicity (referred to herein as the pathogenicity gradient) at multiple doses and four time points in the context of a murine infection model. This allows us to identify pathogenicity-related traits with greater certainty than in previous studies. We utilize global transcriptomic and proteomic data from these experiments, thus providing a more complete view of the layered interaction between host and virus. We demonstrate a network-based approach for identifying critical factors in influenza pathogenesis and test our findings with a pharmacological inhibitor during lethal and non-lethal infections.

## Materials and Methods

### Data Deposition

Microarray data was deposited previously in the gene expression omnibus (GEO) under the following accession numbers: GSE33263: Influenza A/VN/1203/04 infection in mice with three viral doses at 1, 2, 4, and 7 days (IM001); GSE37572: HA avirulent mutation in A/Vietnam/1203/2004(H5N1) infection in mice at 10^4^ PFU at 1, 2, 4, and 7 days (IM004); GSE43301: Influenza A/VN/1203/04 PB2-627E mutant infection in mice with 10^4^ PFU at 1, 2, 4, and 7 days (IM005); GSE43302: Influenza A/VN/1203/04 PB2-627E mutant infection in mice with 10^3^ PFU at 1, 2, 4, and 7 days (IM005); GSE44441: Influenza A/VN/1203/04 PB1-F2 mutant infection in mice with 10^4^ PFU at 1, 2, 4, and 7 days (IM006); GSE44445: Influenza A/VN/1203/04 NS1trunc124 mutant infection in mice with two doses at 1, 2, 4, and 7 days (IM007); GSE37569: Influenza A/CA04/2009 infection in mice with four doses at 1, 2, 4, and 7 days (CA04M001); GSE33266: SARS-CoV MA15 infection in mice with four viral doses at 1, 2, 4, and 7 days (SM001); GSE50000: SARS-CoV MA15, icSARS-CoV, or SARS BatSRBD infection in mice with two viral doses at 1, 2, 4, and 7 days (SM003); GSE49262: SARS-CoV MA15 or SARS deltaORF6 infection in mice with 1O5 PFU at 1, 2, 4, and 7 days (SM012); GSE49263: SARS-CoV MA15 or SARS nsp16 infection in mice with 10^5^ PFU at 1, 2, 4, and 7 days (SM014). Proteomics data for IM001, IM004, IM005, IM006, and IM007 (described above) can be found at https://omics.pnl.gov/project-data/systems-virology-contract-data.

### Construction of Pathogenicity Profile

**FIGURE 1:**
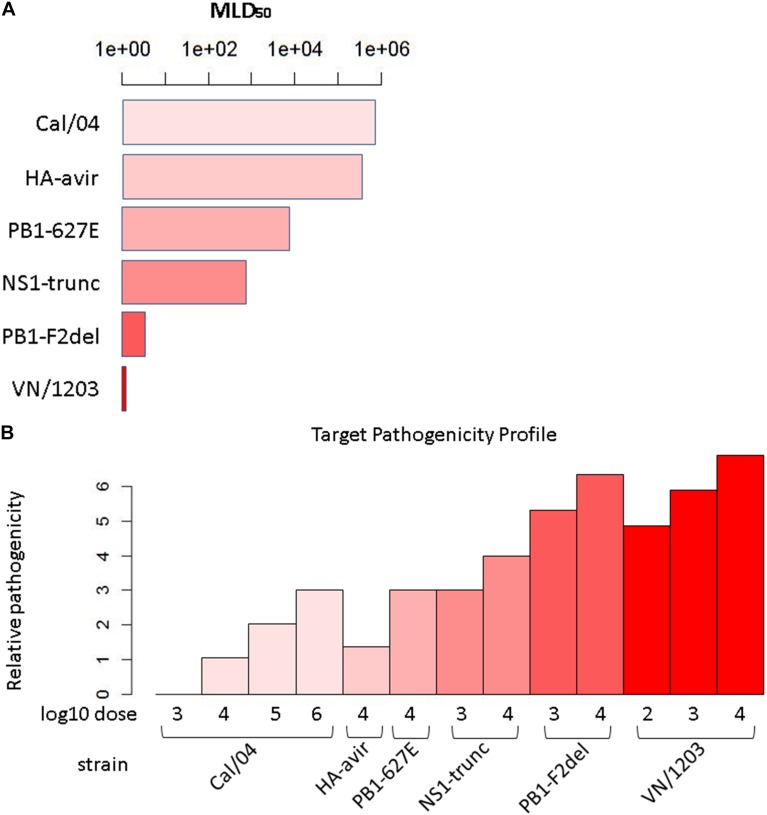
Target pathogenicity profile. **(A)** Median lethal dose 50 (MLD50) values for the six strains/mutants in the influenza virus pathogenicity gradient. Mouse MLD_50_ data was previously published in Tchitchek et al. (2013). **(B)** Target pathogenicity profile based on MLD50 values.

We set out to build a synthetic pathogenicity profile that represents the severity of the infection for each experimental condition. The viruses used to construct the pathogenicity gradient include the H1N1 strain, A/California/04/2009 (CA04), from the 2009 pandemic,; the highly pathogenic H5N1 avian strain, A/Vietnam/1203/2004 (VN1203); and four mutants of VN1203: VN1203-HAavir (which lacks the multi-basic cleavage site in the viral hemagglutinin protein that is critical for extra-pulmonary viral spread), VN1203-PB2-627E (which lacks a mammalian-adapting mutation that substantially increases the replicative ability of the viral polymerase complex in mammalian cells), VN1203-NS1trunc (which encodes a C-terminal truncation in the effector domain of the NS1 host response antagonist protein), and VN1203-PB1F2del (which lacks expression of the PB1-F2 protein). Genes that mirror this profile are hypothesized to be related to pathogenicity in some way, a proposition similar to that given by Taylor et al. (2016). To construct the profile, we scaled all six strains/mutants proportional to their median lethal dose (MLD_50_) value, or the amount of viral particles at which 50% of infected mice succumb to infection (Figure 1A). We therefore assigned a score for each strain corresponding to the log of the MLD_50_ and then adjusted the scores to account for differences in administered doses across studies. Since infection conditions for each strain included a dose at 10^4^ PFU, the corresponding strain’s log MLD_50_ was assigned to all infection conditions at this dose. To make the score more intuitive (high score = high pathogenicity), each log MLD_50_ was subtracted from the maximum observed log MLD_50_. The intent was to quantitatively relate the experimental conditions to each other, with the expectation that genes related to pathogenicity would manifest expression patterns similar to the pathogenicity profile. To avoid negative values, an additional unit was added to each score. Therefore, the pathogenicity level for a given infection condition *i*, with dose *d*_*i*_ and the particular viral strain’s MLD_50_ *m*_*i*_, is given by:

$$P_{i}=1+\log\left(\frac{m_{m\,a\,x}}{m_{i}}\right)+\log\left(\frac{d_{i}}{d_{c\,o\,m}}\right)$$

where *m*_*max*_ is the maximum observed MLD_50_, and *d*_*com*_ is the dose common to all strains/mutants, i.e., the experimental conditions for all infections included at least this dose, if not others. Applying this calculation across all conditions yielded the profile (Figure 1B). Since the array of conditions differed somewhat between the transcripts and protein data, profiles unique to each of these datasets were generated.

### Data Pre-processing

Sample collection and microarray processing are described for this dataset in Tchitchek et al. (2013). For our analysis, we selected the probes that were (1) present on all arrays after quality control filtering, (2) previously identified as significantly changed from mock expression (*q*-value < 0.05), and (3) had a simultaneous log2 fold change of at least 1.5 in at least one experimental condition (Li et al., 2011). This resulted in the selection of 7471 probes for analysis. The results for proteomic data, sample processing, capillary LC-MS/MS analysis, spectral matching, and peptide-to-protein rollup are described in Tchitchek et al. (2013). Missing value imputation was performed using a regularized expectation maximization algorithm (Webb-Robertson et al., 2015). Selected proteins were only those that were (Peiris et al., 2004) detected in every experiment before imputation (experiment = set of infections with one given strain), and (Food and Agriculture Organization [FAO], 2019) significantly changed from mock (*p* < 0.05) in at least one experimental condition; this resulted in 1476 proteins that were used in our analysis.

For the correlation calculations (below), expression data from selected time points were extracted and then mean-summarized for each strain and dose. Some viruses received heavier experimental coverage than others; we therefore expanded the data compendium to equalize the influence each virus strain in the pathogenicity gradient has on the correlation calculations by duplicating the data from under-represented conditions so that every strain was equally represented in the compendium.

### Correlation

We calculated Pearson’s correlation using the expanded expression profile for each gene/protein and the appropriately expanded pathogenicity profile. Day 1, day 2, day 4, and day 7 designations were used to refer to the top 5% of pathogenicity-correlated genes (both positive and negative correlation) using individual time point data for correlation calculations.

### Association Network Topology

Network inference was performed using the Context Likelihood of Relatedness software tool as described (Mitchell et al., 2013). The network centrality measures of betweenness and degree were determined using the R igraph package.

### Interactor Enrichment Analysis

To identify regulators of interest, we used the “Interactions By Protein Function” and “Significant Interactions Within Set(s)” tools in the MetaCore software package (Clarivate Analytics, Philadelphia PA) to identify genes/proteins whose interactors were enriched among pathogenicity-related proteins. A similar approach was used for pathogenicity-related genes.

### Cluster Analysis

We used the R weighted gene correlation network analysis (WGCNA) package to identify clusters of genes or proteins with behavioral similarity (Langfelder and Horvath, 2008). Cluster identification was performed using the blockwiseModules function with the following parameter values for transcript cluster analysis: power = 12, minModuleSize = 30, maxBlockSize = 8000, reassignThreshold = 0, mergeCutHeight = 0.25, and pamRespectsDendro = F.

### Ethics Statement

All animal experiments and procedures were approved by the University of Wisconsin (UW)-Madison School of Veterinary Medicine Animal Care and Use Committee under relevant institutional and American Veterinary Association guidelines.

### Biosafety

All experiments using replication competent H1N1 viruses were performed in biosafety level 2 (BSL-2) or animal enhanced biosafety level 2 (ABSL-2) containment laboratories at UW-Madison. Experiments using replication competent H5N1 virus were performed in an ABSL-3+ containment laboratory at UW-Madison. UW-Madison BSL-2, ABSL-2, and ABSL-3+ laboratories are approved for use by the United States (US) Centers for Disease Control and Prevention (CDC) and the US Department of Agriculture. Experiments using replication competent SARS-CoV were performed in an ABSL-3+ containment laboratory at the University of North Carolina at Chapel Hill (UNC). UNC BSL-2, ABSL-2, and ABSL-3+ laboratories are approved for use by the US CDC.

### Cells

Madin-Darby canine kidney (MDCK) cells were propagated in a minimum essential medium (MEM) containing 5% newborn calf serum and were maintained at 37°C in an atmosphere of 5% CO_2_. Cell stocks are periodically restarted from early passage aliquots and routinely monitored for mycoplasma contamination.

### Viruses

The A/California/04/09 H1N1 virus (CA04) was provided by the US CDC. The A/chicken/Vietnam/TY167/2011 (H5N1) virus (TY167) was obtained through surveillance activities in Vietnam. Stock viruses were generated by passaging an aliquot of the original virus once in MDCK cells containing 0.6% bovine serum albumin (BSA) fraction V (Sigma-Aldrich) and 1 μg/ml tosyl phenylalanyl chloromethyl ketone (TPCK)-treated trypsin (CA04) or in embryonated chicken eggs (TY167), as previously described (Eisfeld et al., 2014). Stock virus titers were quantified by plaque assay in MDCK cells using standard methods. SARS-CoV was propagated and assayed for titer levels, as published previously (Gralinski et al., 2013).

### Mouse Infections

Nine- to ten-week-old female C57BL/6J mice (The Jackson Laboratory) were administered 0 or 100 mg/kg of Gefitinib (Tocris Bioscience) in 1% Tween-80 in phosphate-buffered saline (PBS) by oral gavage 1 day prior to infection and each subsequent day until the end of the experiment. Four mice were used for each drug and viral dose combination. For infection, mice were anesthetized by intraperitoneal (i.p.) injection of ketamine and dexmedetomidine (45–75 mg/kg ketamine + 0.25–1 mg/kg dexmedetomidine) and were intranasally inoculated with 50 μl of PBS-containing viruses, as indicated in Figure 6 and the corresponding text in the “Effect of EGFR Inhibition on Influenza Pathogenesis in Mice” section. Following inoculation, dexmedetomidine was reversed by i.p. injection of atipamezole (0.1–1 mg/kg). Subsequent to infection, individual body weights and survival were monitored for up to 17 days, and mice were humanely euthanized when exhibiting severe clinical symptoms or at the end of the observation period. SARS-CoV infections in mice were performed as previously published (Gralinski et al., 2013).

### Statistical Analysis

To model the trend in mice weight over time, we used linear mixed effects models with a normal conditional distribution and identity link on each time course. Fixed effects were day, gefitinib level, and the interaction between day and gefitinib level, while random effects for each mouse were included to account for variability in the mice and for the non-independent nature of the data over time. A second model that did not include the gefitinib level and day interaction terms was also fit; a likelihood ratio test was then conducted to determine if the group slopes were significantly different.

Due to the patterns of mouse weight over time, a single linear model was not always sufficient to model the data; this was determined by using linear splines to estimate change points where separate linear models should be used to represent the trend for different time ranges. Specifically, knot points were identified by determining if adding an additional time point to the linear model of an existing segment changed the model. If the new point changed the model significantly, then a new knot point was identified; otherwise, the time point was added to the segment. After all knot points were found, a single random mixed effect model was fit to each segment of data. TY167 at 10^3^ PFU was modeled as a single segment, CA04 at 10^2^ PFU and 10^3^ PFU in three segments, and TY167 at 10^2^ PFU in four segments. Separate *p*-values were determined for each segment (as identified from the knot points as boundaries), which are provided in Table 2.

## Results

### Experimental Overview

**FIGURE 2:**
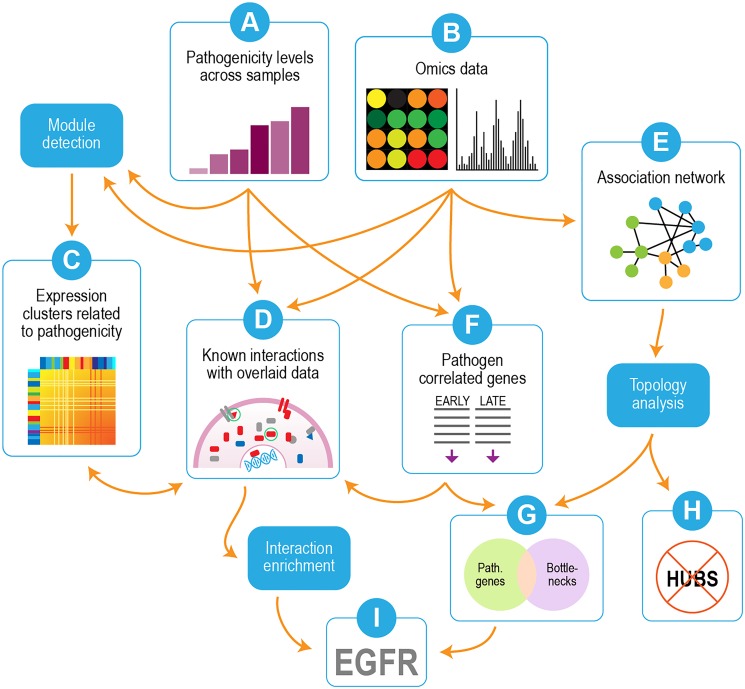
Overview of analysis strategy. Omics data (transcriptomics and proteomics) were used in conjunction with pathogenicity data from the different virus strains/mutants **(A,B)**. Correlated modules in the transcriptomics were detected **(C)** and compared with the pathogenicity profile **(A)** to identify gene modules whose behavior linked them to pathogenicity. Individual proteins whose behavior correlated with pathogenicity were submitted to interaction enrichment analysis, which looked for genes whose interaction neighbors from curated networks were enriched among pathogenicity-correlated proteins **(D)**. An association network built from mutual information of perturbed gene pairs **(E)** was used for topology analysis, which yielded network hubs and bottlenecks. Lists of pathogenicity-correlated genes from early and late time points **(F)** were combined and compared to network nodes with high hub and bottleneck scores. Overlap was seen with network bottlenecks **(G)** but not hubs **(H)**. EGFR was identified as a candidate for follow-up experiments based on overlaps between interaction enrichment and pathogenicity-related bottlenecks **(I)**.

Our overall strategy is depicted in Figure 2. We used transcriptomics and proteomics data in conjunction with pathogenicity data from the different virus strains/mutants (Figures 2A,B) to identify pathways and individual genes/proteins that were important for influenza pathogenicity. Correlated gene modules in the transcriptomics were first detected (Figure 2C) and compared with the pathogenicity profile (Figure 2A) to identify gene modules whose behavior linked them to pathogenicity. Individual proteins whose behavior correlated with pathogenicity were incorporated into interaction enrichment analysis, which identified genes whose interaction neighbors from curated networks were enriched among pathogenicity-correlated proteins (Figure 2D). These results could be connected to host responses evident in the gene clusters depicted in Figure 2C. An association network built from mutual information of perturbed gene pairs (Figure 2E) was used for topology analysis, which yielded network hubs and bottlenecks. Lists of pathogenicity-correlated genes from early and late time points (Figure 2F) were combined and compared to network nodes with high hub and bottleneck scores. Comparison of genes correlated with pathogenicity to network hubs and bottlenecks showed significant overlap with network bottlenecks (Figure 2G), but hubs were strikingly excluded (Figure 2H). Examination of results from interaction enrichment (Figure 2D) and network topology (Figures 2E–G) revealed epidermal growth factor receptor (EGFR) as a candidate for follow-up experiments (Figure 2I).

### Correlation With Pathogenicity

We utilized transcriptomic and proteomic datasets (represented by Figure 2B) of mouse infection with six strains/mutants of influenza at varying doses and times, as described in Tchitchek et al. (2013). These viruses display varying degrees of virulence, as assessed by the minimal dose that is lethal to 50% of animals to which it is administered (MLD_50_, Figure 1A, see section “Materials and Methods” for details). Samples were collected at 1, 2, 4, and 7 days post-infection for global transcriptomics and proteomics analysis of lung tissues relative to time-matched mock-infected controls.

### Clustering of Expression Data

**FIGURE 3:**
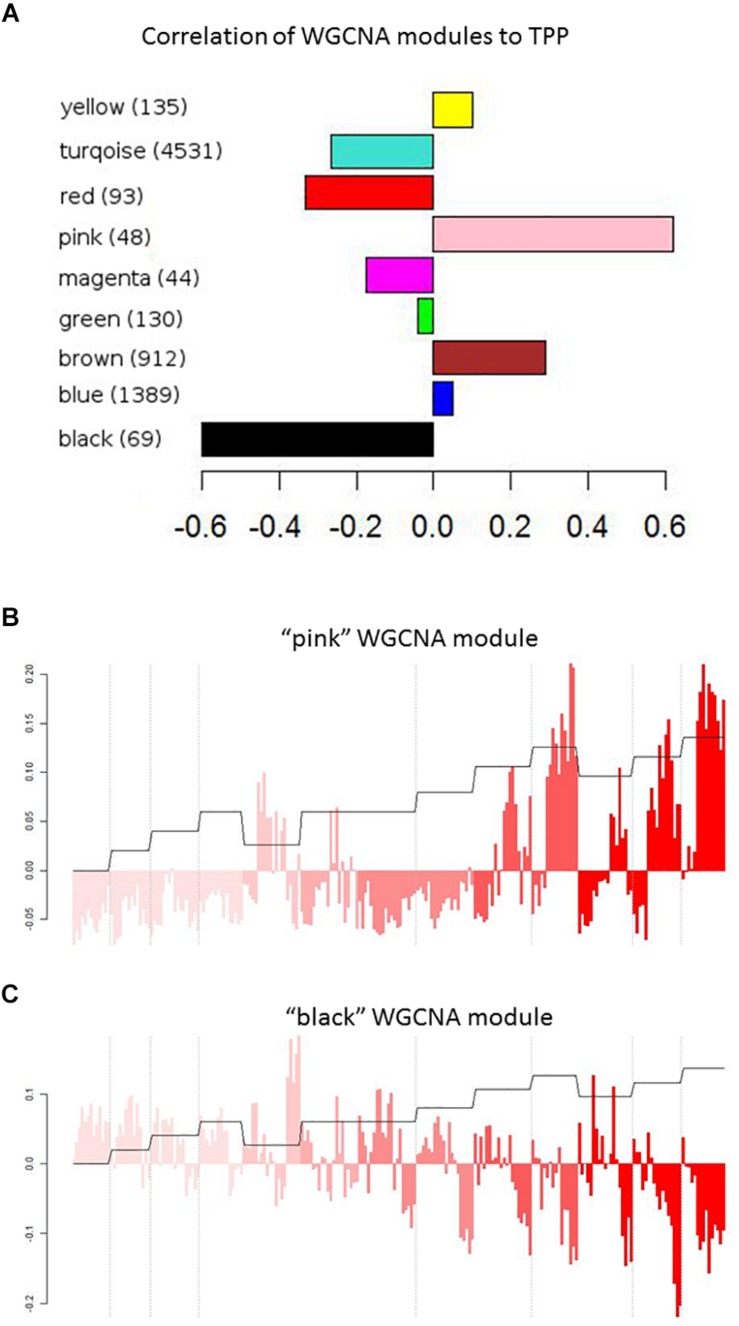
WGCNA modules. **(A)** Pearson’s correlation values of WGCNA modules with the pathogenicity profile. **(B,C)** Correlation of the “pink” **(B)** or “black” **(C)** WGNCA module eigengene with the pathogenicity profile. Module eigengenes are the principle component of the expression levels of modules that contains similarly behaving genes, and therefore represent the expression patterns across all genes in a module. Each bar represents the expression of the module’s genes in an individual mouse infected with a particular strain of influenza at a certain dose for a set number of days (1, 2, 4, or 7). Color intensity represents strains/mutants with increasing pathogenicity, as in Figure 2. Dashed lines indicate dose separations for a single strain. Orange trace shows the pathogenicity profile.

To identify transcripts correlated with pathogenicity, we used the WGCNA network clustering approach (Langfelder and Horvath, 2008) to cluster gene expression profiles across all experiments into expression modules that represent groups of genes with similar expression behaviors (Figure 2C). This approach further affirms that the overall gene expression pattern of each module has true biological meaning because each identified pattern is manifested by many genes. The representative expression profile of each module, or eigengene, can be correlated with clinical measures or other metadata to identify modules of interest (Langfelder and Horvath, 2008; Saris et al., 2009; Levine et al., 2013). We thus applied WGCNA to our transcript dataset to identify network modules related to influenza pathogenicity. Figure 3A shows the correlations of all eigengene profiles to pathogenicity. Two of the modules, pink and black, showed much higher correlations than the others and were selected for further analysis. The pink module is strongly *positively* correlated with pathogenicity (Figure 3B). We found statistical enrichment in plasminogen activation among the genes in this module, particularly KLKB1 and coagulation factor X1; this suggests that pathogenic influenza infection involves a perturbed coagulation cascade. The black module (Figure 3C) is strongly *negatively* correlated with pathogenicity and was strikingly enriched for B-cell activation, implying that a diminished presence of B-cell activity is related to pathogenesis in influenza. Interestingly, a previous report showed that influenza caused apoptotic loss of bone marrow B-cells in mice despite the complete lack of viral particles detected in the bones (Sedger et al., 2002). Since B-cells are known to both reside in and travel through the lungs (Polverino et al., 2016), high-path flu may trigger death of lung B-cells, resulting in previously unappreciated effects on host response during these infections.

### Transcriptome/Proteome Integration

To determine the extent to which proteome data validated our transcriptomics findings, we used the matching protein expression data to evaluate downstream pathway regulation related to infection severity at different time points (Figure 2D). We reasoned that correlation with pathogenicity could have different meanings depending on which time points are used for the correlation calculation. Correlated genes identified early in the infection could be filling regulatory roles, while those from later points are expected to be the downstream effects of earlier events. Accordingly, we identified genes and proteins whose expression profiles correlated with pathogenicity at individual time points and designated the resulting lists as day 1, day 2, day 4, and day 7. We then integrated the gene and protein data using target enrichment to identify the regulatory targets of protein pathway expression (Supplementary Tables S1, S2). We found that Syk, Prkcb, and Ebf1 were day 1 genes and that each of these is a regulator of B-cell activation/maturation. Proteins known to be regulated by and/or bind to these regulators were significantly enriched among day 1 (Syk) proteins, while transcripts of genes regulated by Ebf1 and Prkcb were enriched among day 1 and day 7 (Ebf1) or day 4 (Prkcb) genes. Like the B-cell-related module from the cluster analysis, the expression profiles of all three of these B-cell regulators showed strong negative correlation to pathogenicity, thus reinforcing the concept of decreased B-cell presence in the mouse lung during severe influenza infection. Thus, our observation that the presence of B-cell-related functions is tied to pathogenicity is borne out by comparing results across time points and data types. Similarly, we observed enrichment in proteins regulated by coagulation factor XIII A1 (F13a1) in day 4 proteins, with the F13a1 transcript also among day 4 genes. Transcripts for coagulation regulators Plat and Serpine1 as well as their downstream targets were found among day 7 genes. These latter results validated our findings from transcript expression that coagulation-related pathways are linked to pathogenicity. In addition to findings related to regulation of B-cell activity and coagulation, we also observed that direct protein targets of EGFR activity are significantly enriched among day 1 and day 4 proteins. In this way, integration of transcriptomic and proteomic data enhances our analysis and identifies the pathways most likely to be important for infection severity.

From this analysis we constructed three lists of genes that were identified as being correlated to pathogenicity in early infection (Supplementary Table S3), late infection (Supplementary Table S4), or both (Figure 2F and Supplementary Table S5). We focused on genes that are correlated with pathogenicity both early and late in the infection, for two reasons: (Peiris et al., 2004) high correlations from two separate groups of data points for the same gene means it is likely that these genes truly correlate with pathogenicity, and (Food and Agriculture Organization [FAO], 2019) the overlap of the two groups helps yield the identity of genes important both early and throughout the infection process. Because influenza viral titers reach maximal levels by day 2, regulatory responses are likely to occur in the first 24 h of infection. We thus designated the day 1 results as “early” and other time points as “late.” To identify genes with both early and late infection correlation to pathogenicity, genes were identified from the top 5% of pathogenicity-correlated genes at early time points. The same procedure was used for late time points, and the intersection of these two resulted in a list of early/late correlated genes. The overlap between day 1 genes and the set of combined day 2, day 4, and day 7 genes resulting in 54 genes (we refer to these as early/late correlated genes). The overlap between early and late was highly significant (*p* = 6.4e-13, Fisher test).

### Association Network Topology Analysis

The clustering analysis provided a way to determine what kinds of genes manifested expression behaviors connected to pathogenicity. However, we were interested in identifying regulatory mechanisms of influenza infection in the context of pathogenicity. As a means of identifying key regulators, we turned to an approach based on network topology. A growing body of work has shown that network topology, or the placement of nodes in the network structure, can be used to identify entities with key regulatory roles (Yu et al., 2007; Zhou and Liu, 2014; Narang et al., 2015; McDermott et al., 2016). Of particular interest are network bottlenecks and hubs, both of which have been shown to be enriched for regulators under various circumstances (Yu et al., 2007; McDermott et al., 2009, 2012, 2016; Zhou and Liu, 2014; Narang et al., 2015). We built a mutual information-based association network with transcript data (McDermott et al., 2009, 2016; Mitchell et al., 2013) using 7471 genes deemed significantly changed in at least one experimental condition (strain, dose, or time; see section “Materials and Methods”) as input; we also identified network hubs and bottlenecks, which were defined as the top 5% of betweenness scores and degree scores, respectively (Figure 2E).

**FIGURE 4:**
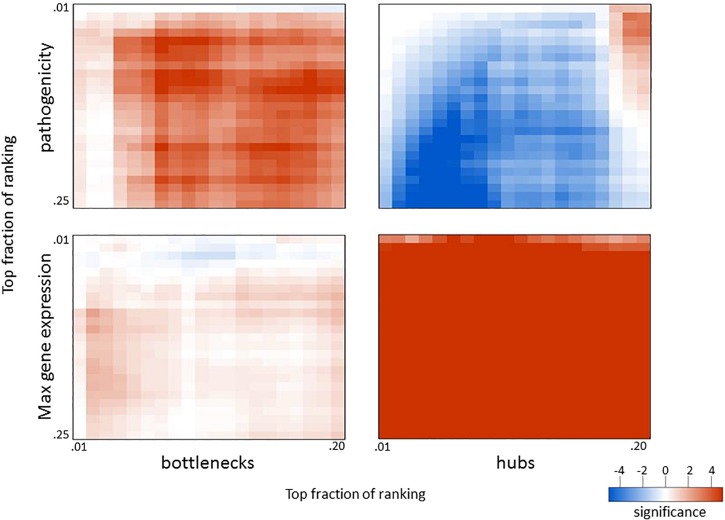
Overlap in biological measures and graph topology for influenza infection. Genes were ranked according to their correlation with the pathogenicity profile (**top** panels), maximum fold change across all infection conditions (**bottom** panels), network betweenness (**left** panels), and network degree (**right** panels). The top fraction from one ranking was compared to the top fraction in the other ranking using a two-tailed Fisher’s exact test as indicated. Numerical scale represents the absolute value of the log10 *p*-value. For negative enrichment, these values were multiplied by –1.

Since network hub nodes are known to have critical systemic functions, we hypothesized that pathogenicity-related genes may be enriched in bottleneck or hub genes. To test this hypothesis, we examined the statistical enrichment of the identified pathogenesis sets with bottlenecks and hubs identified from the network analysis. Interestingly, no overlap was discovered between network hubs and early/late correlated genes (*p* = 0.11, two-sided Fisher test). In contrast, we found these genes to be significantly enriched in network bottlenecks (10 of the 54, *p* = 2.9e-4, two-sided Fisher test), suggesting bottlenecks are important early regulators of pathogenesis (Figures 2G,H; names and descriptions of the genes are found in Table 1). Notably, three of these ten, CD22, FCRL1, and IKZF3, are closely related to B-cell activation and overlapped with members of the black WGCNA module. Another of these 10 genes is EGFR (Figure 2I), which we found to have protein targets enriched among correlated proteins at day 1 and day 4. To determine if these results were biased by the selection of arbitrary thresholds, we generated rankings for the degree, betweenness, and correlation to pathogenicity of all genes, then produced matrices of upper percentile thresholds by applying a Fisher enrichment test for each threshold pairing. Remarkably, we observed a dramatic exclusion (blue cells in Figure 4, upper right) of hubs from correlated genes across a wide range of thresholds. In contrast, bottlenecks showed a strong enrichment trend (red cells in Figure 4, upper left). These results show that for influenza infection, network hubs and bottlenecks have strikingly opposite roles regarding pathogenicity of the virus.

**TABLE 1:** Pathogenicity-related bottleneck genes.

**Gene symbol**	**Entrez**	**Description**	**Comment**
EGFR	13649	Epidermal growth factor receptor	Receptor tyrosine kinase
TBC1D10C	108995	TBC1 domain family, member 10c	Inhibits Ras and calcineurin
CD22	12483	CD22 antigen	B-cell/B-cell interactions
FCRL1	229499	Fc receptor-like 1	Ig receptor, promotes B-cell activation and differentiation
ELOVL1	54325	Elongation of very long chain fatty acids	Associated diseases include peroxisomal disease and adrenoleukodystrophy
IKZF3	22780	IKAROS family zinc finger 3	B-cell activation and differentiation
MBD1	17190	Methyl-CpG binding domain protein 1	Transcriptional repressor of methylated DNA
TSPAN32	27027	Tetraspanin 32	Involved with hematopoietic cell function, associated with some cancers
KIF21B	16565	Kinesin family member 21B	ATP-dependent microtubule-based motor protein, associated with inflammatory bowel disease and multiple sclerosis
SLC10A6	75750	Solute carrier family 10 (sodium/bile acid cotransporter family), member 6	Lung sulfonated steroid importer

Given that network bottlenecks have a unique relationship with genes related to pathogenicity, we hypothesized that network hubs might be enriched in genes involved in more general aspects of infection. To test this, we identified the most highly perturbed genes across the transcriptome by ranking genes by their maximum fold change value across all data sets and then built matrices that compared the maximum expression to betweenness and degree, as before. As shown in the lower panels of Figure 4, genes with high maximum expression overlapped dramatically with network hubs but showed minimal enrichment for bottlenecks. Thus, highly connected genes (hubs) are strongly related to high expression and are strongly segregated from pathogenicity-related genes, while network bottlenecks show a strikingly different strong relationship to pathogenicity.

**FIGURE 5:**
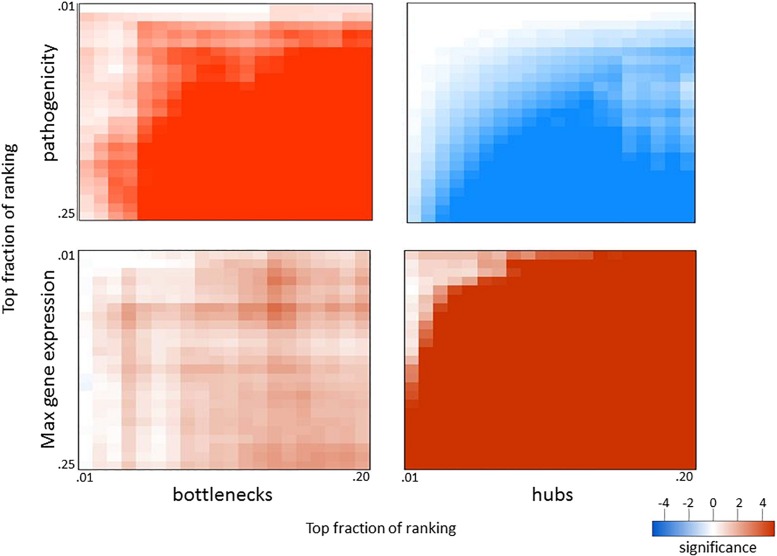
Overlap in biological measures and graph topology for SARS-CoV infection. Significance matrices were generated for SARS-CoV infection experiments as in Figure 4; however, weight loss values at each infection condition were used as a pathogenicity measurement. Weight loss correlations for early (days 1 and 2) and late (days 4 and 7) were combined to obtain the pathogenicity ranking.

To determine if similar relationships exist in a distinct infection system, we applied a similar analysis to a compendium of four datasets obtained from mice infected with the SARS coronavirus (SARS-CoV), one of which was previously published by Gralinski et al. (2013). Mice were infected with WT SARS-CoV and three attenuated mutants at varying doses and analyzed for lung gene expression at one, two, four, and seven days post-infection. Since lethality is not readily observed in attenuated SARS-CoV mutants, we used animal weight loss at each time point to represent pathogenicity at each infection condition. Also, since viral replication kinetics are slower for SARS-CoV infection compared to that of influenza virus, we used days 1 and 2 to represent early infection and days 4 and 7 for late infection when ranking genes for pathogenicity. When applying the approach outlined above, we observed very similar results with SARS-CoV to those seen with influenza virus (Figure 5). The same patterns of exclusion and enrichment of hubs and bottlenecks in regard to pathogenesis-correlated genes and high-expression genes was shown to be even more dramatic in SARS-CoV. Thus, the enrichment of pathogenicity-related genes in network bottlenecks and their exclusion from network hubs appears to be a widespread phenomenon characteristic of respiratory viral infections in mice.

This finding is significant because the network betweenness measurement we applied was in no way informed by our pathogenicity results, yet it is able to significantly enrich for pathogenicity-related genes. Thus, network bottlenecks but not hubs facilitate the identification of critical regulators as intervention targets. Further studies will determine whether this approach is applicable in other infection systems as well.

Interestingly, no overlap was found between pathogenicity-related genes in influenza and SARS-CoV, but significant overlap in bottlenecks (39 genes, *p*-value < 10^–6^) and hubs (203 genes, *p*-value << 10^–6^) was found between the two viruses.

### Effect of EGFR Inhibition on Influenza Pathogenesis in Mice

**FIGURE 6:**
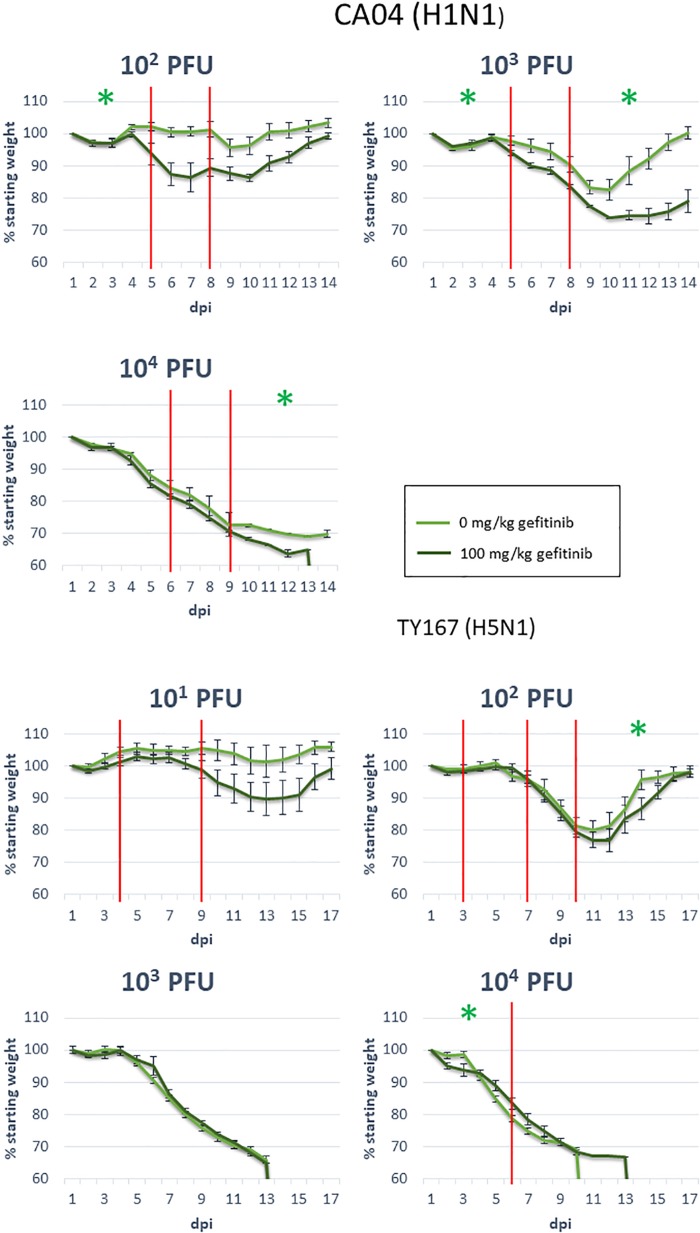
EGFR inhibition during influenza infection. Mice were exposed to the indicated dosages of gefitinib and influenza virus strains, then monitored for body weight over the indicated days post-infection (dpi). Red vertical lines indicate knot points for linear modeling (see sections “Materials and Methods” and “Statistical Analysis”). Green star: significance below 0.05; see Table 2 for segments with near-significant changes (segments with no significance indication had *p*-values above 0.1).

Epidermal growth factor receptor has previously been shown to play a role in influenza infection (Eierhoff et al., 2010; Ueki et al., 2013) but has not been tied to pathogenicity. Because we identified EGFR as a pathogenicity-correlated bottleneck gene with apparent signaling effects evident in proteomics and transcriptomics, we investigated the role of EGFR in pathogenesis further with a mouse model. We treated mice for 14 days with the EGFR inhibitor gefitinib and monitored infection-related weight loss of treated and untreated animals to determine if EGFR inhibition affected the course of infection. After one day of treatment, mice were infected with one of two strains of influenza virus at various doses: CA04 (10^2^, 10^3^, and 10^4^ PFU) or the highly pathogenic H5N1 avian strain, A/chicken/Vietnam/TY167/2011 (TY167) (10^1^, 10^2^, 10^3^, and 10^4^ PFU). The alternative H5N1 strain was used since the MLD50 for VN-1203 is very low and makes identification of drug effects difficult. Linear mixed effects models were used to model the weight loss trajectories from different infection conditions and to determine if the weight loss slope differed between treatments (Table 2 and Figure 6). Red lines represent segments of the data that could be modeled with a single linear model; segments are separated by knot points. For all doses of Cal/04 and the two lower doses of TY167, drug treatment significantly increased infection severity in some segments. However, higher doses of TY167 erased this trend and possibly partially reversed it. While all animals died by day 13, weight loss was less rapid by a small but significant margin in drug-treated animals at the highest viral dose. Thus, EGFR appears to play a significant role in the severity of non-lethal infections such that when it is inhibited, the infection is more severe. However, when the threshold is crossed to the highly lethal pathogenesis of H5N1, other mechanisms potentially take over and supersede or override the role of EGFR.

**TABLE 2:** Modeling strategy for weight loss results from EGFR inhibition study.

**Experiment/**		**Knot**	
**Viral load**	**Approach**	**point**	**Results: *p*-value**
CA04/10^2^ PFU	Data modeled in 3 segments	Day 5	Segment 1: 0.01979
		Day 8	Segment 2: 0.7377
			Segment 3: 0.07305
CA04/10^3^ PFU	Data modeled in 3 segments	Day 5	Segment 1: 0.01059
		Day 8	Segment 2: 0.7170
			Segment 3: 0.002228
CA04/10^4^ PFU	Data modeled in 3 segments	Day 6	Segment 1: 0.3498
		Day 9	Segment 2: 0.9064
			Segment 3: 0.03589
TY167/10^1^ PFU	Data modeled in 3 segments	Day 4	Segment 1: 0.09511
		Day 9	Segment 2: 0.2128
			Segment 3: 0.8031
TY167/10^2^ PFU	Data modeled in 4 segments	Day 3	Segment 1: 0.4661
		Day 7	Segment 2: 0.6107
		Day 10	Segment 3: 0.2895
			Segment 4:.03364
TY167/10^3^ PFU	Data modeled in 1 segment	NA	0.074
TY167/10^4^ PFU	Data modeled in 4 segments	Day 6	Segment 1: 0.02235
			Segment 2: 0.0769

## Discussion

We used a multi-faceted approach to uncover critical components of pathogenicity in an attempt to take full advantage of the pathogenicity gradient in our study’s influenza viruses and mutants. We compared the expression behavior of genes and proteins to the pathogenicity measurements of viruses in our study; this allowed us to identify which pathways and features are most closely associated with pathogenicity. The results provide clues to the underlying causes of the severity of highly pathogenic strains.

Our group previously used this dataset to determine that host responses to various infection conditions involve similar pathways but are characterized by distinct kinetic expression profiles (Tchitchek et al., 2013). In the current study, we use a complementary approach to identify the genes and pathways that are most closely associated with more pathogenic viruses instead of identifying elements common to all. We show that the network topology of association networks can be used to predict genes’ involvement in pathogenicity-related processes. We used this knowledge in conjunction with other network methods to identify genes and pathways associated with disease severity. Our results show that signaling downstream of EGFR, coagulation pathways, and B-cell down-regulation in the lung are tied to infection severity in highly pathogenic influenza. A follow-up validation study in mice confirms the role of EGFR in influenza pathogenicity.

We first asked what broad trends in pathogenicity could be identified using a network clustering approach. One of the detected network clusters was strongly enriched for functions related to B-cells and was negatively correlated with the pathogenicity profile. This could be caused by either a general down-regulation of gene expression in lung B-cells or a general loss of B-cells from the lung. Although influenza may infect B-cells expressing flu-specific B-cell receptors (Dougan et al., 2013), initially naïve mice from our experiment are not likely to have expanded virus-specific B-cells during the time frame of our OMICs experiments. Thus, the effect is not likely a result of gene regulation within infected B-cells and is more likely due to a diminished B-cell lung population in highly pathogenic infections. A previous report demonstrated apoptotic death of bone marrow B-cells in flu-infected mice despite failing to show that the virus was present in the bone marrow (Sedger et al., 2002). Therefore, a systemic signal appears to target remote B-cells and may target lung B-cells as well. While the adaptive immune response is not likely to play a direct role during the time frame of these experiments, a lower B-cell population, for whatever reason, may signal important immune response dynamics not previously understood. Histological or other studies would be necessary to confirm the relationship between severe infection and diminished B-cell numbers. The second cluster was related to coagulation/fibrinolysis, which has shown a precedent in previous work for an involvement in influenza infection (Berri et al., 2013). Plasminogen (which opposes clot formation) appears to promote destructive inflammation during influenza infection. While we observed both pro- and anti-coagulation factors that were positively correlated with pathogenicity, these responses may represent a mixture of virus-induced responses and host responses to a pathogenic state.

We then corroborated these results by identifying links between pathogenicity-correlated genes and pathogenicity-correlated targets of these genes. Since a dataset of this kind deals strictly with the expression of genes and proteins, other events such as protein–protein interactions, protein–mRNA interactions, and phosphorylation/dephosphorylation events are not directly monitored. Thus, a portion of the very early events that determine the severity of an infection is not observable with this dataset. By integrating transcript and protein data, however, we were able to reveal links between upstream and downstream effectors for EGFR signaling, coagulation regulation, and B-cell down-regulation that would not be possible without the availability of both data types. Since correlations between transcript and protein expression profiles are consistently observed to be low across biological systems (Vogel and Marcotte, 2012), validation of transcript expression using direct correlation of protein abundances is generally not successful. However, we believe that functional rather than direct correspondence of transcripts and proteins represents an effective integration of both data types. Hence, our study provides hypotheses for the involvement of a number of genes/pathways in the pathogenicity of influenza virus.

To learn more about regulatory mechanisms of influenza infection, we determined whether topological positions in association networks were related to pathogenicity. We found that genes correlated with pathogenesis overlapped significantly with bottlenecks but were dramatically excluded from hubs. This result may be explained by the fact that network hubs are highly connected to many other network nodes so that rather than being involved only in highly pathogenic conditions, they tend to be involved in *all* infection conditions. This is affirmed by the observation that genes with the largest changes in gene expression (therefore likely to exert the strongest influence on other genes) were very strongly enriched for hub genes. On the other hand, network bottlenecks represent nodes linking different areas of the network and may identify genes that have an influence on only a subset of the processes being monitored by the data. Interestingly, in a network built with data from infections of varying pathogenicity, the genes exerting these influences appear to be involved in pathogenicity-related processes. The same relationships between network topology, viral pathogenicity, and gene expression that were observed for influenza virus were also noted when we used a similar dataset of SARS-CoV infections, thus further validating our analysis and demonstrating that these relationships appear to apply to respiratory viruses in general. We observed remarkably high (77% of possible) overlap between hub genes in SARS-CoV and influenza virus networks; this is consistent with the tendency of these genes to have a universal influence during infectious disease. In contrast, bottlenecks and pathogenicity genes showed much lower or non-existent overlap between the two infection systems, suggesting that each virus maintains unique mechanisms of host interaction. This finding is important because it demonstrates that the identification of non-hub bottlenecks may represent a way to naively identify virus-specific pathogenicity-related genes when pathogenicity data is not available. Previous work has shown that network bottlenecks have important regulatory roles (Yu et al., 2007; McDermott et al., 2009, 2011, 2012, 2016; Mitchell et al., 2013), but this is the first time that an association has been seen between bottlenecks and pathogenesis, with network hubs being conspicuously excluded.

To validate our findings, we treated mice with the EGFR inhibitor gefitinib during infection with high- and low-path influenza. Weight loss was significantly worsened when EGFR was inhibited during low-path infection as well as during low dose infection treatment with a highly pathogenic strain, all of which were non-lethal infections. These results suggest that care should be taken when administering gefitinib to patients at risk of or currently infected with influenza. Interestingly, however, high-dose, high-pathogenicity conditions displayed a possible reversal of this trend, with gefitnib showing a significant slowing of the weight loss trend at the highest dose. Thus, the role of EGFR is dependent on the severity of the current infection, indicating a role in pathogenicity as predicted by our OMICS studies. EGFR stimulation has previously been shown to play a role in promoting influenza particle uptake, and EGFR inhibition diminished viral titer in infected mice (Eierhoff et al., 2010; Ueki et al., 2013). However, the effect of EGFR inhibition on pathogenicity was not determined in previous studies. Viral titer measurements made during this experiment would have allowed us to determine the effect of the drug on viral replication simultaneously with pathogenicity, allowing a clearer picture of the mechanisms at play during EGFR inhibition. While the specific mechanisms are unknown, our results point to a scenario where EGFR inhibition mainly exacerbates pathogenicity at low severity, likely because of the resulting blockage of host benefits such as wound-healing in the lungs (Puddicombe et al., 2000). Interestingly, SARS-CoV infection in the context of overactive EGFR results in pulmonary fibrosis (Venkataraman et al., 2017), supporting the idea that EGFR signaling supports tissue regrowth during respiratory infection. The beneficial effect that comes from preventing viral particle uptake is only apparent under severe conditions when the host is largely unable to repair damaged tissue, as is likely the case in our high-dose, high-pathogenicity infection when mice are moribund. Thus, EGFR activation is a double-edged sword in influenza infection, promoting viral replication through increased virion uptake or suppression of cytokine production (Kalinowski et al., 2014) while simultaneously driving tissue maintenance. This shift in the effect of EGFR inhibition across pathogenicity provides new clues to the role of EGFR regulation during lethal and non-lethal influenza virus disease.

In summary, we have used a unique combination of network-based analyses of transcript and protein expression from our pathogenicity gradient dataset to (1) identify B-cell down-regulation and coagulation pathway up-regulation as being likely associated with pathogenicity in influenza; (2) show that identification of non-hub bottlenecks represents a way to use association networks to enrich prediction of pathogenicity-related genes and pathways; (3) validate the involvement of one of these pathways, EGFR signaling; and (4) show that EGFR inhibition appears to override a key host response mechanism involved in non-lethal viral infections.

## Data Availability Statement

The transcriptomics datasets generated for this study can be found in Gene Expression Omnibus, GSE50000, GSE49262, GSE33263, GSE37572, GSE43301, GSE43302, GSE44441, GSE44445, GSE37569, GSE33266, and GSE49263. Proteomics datasets can be found at https://omics.pnl.gov/project-data/systems-virology-contract-data.

## Ethics Statement

All animal experiments and procedures were approved by the University of Wisconsin (UW)-Madison School of Veterinary Medicine Animal Care and Use Committee under relevant institutional and American Veterinary Association guidelines.

## Author Contributions

ML isolated a virus strain. AE, LG, RB, YK, and KW designed the experiments. AE, LG, and AS performed the experiments. HM, KS, JM, and KM conceived and designed the analysis. HM, KS, NH, and JW performed the analysis. HM and KW wrote the manuscript. All authors reviewed the manuscript.

## Conflict of Interest

The authors declare that the research was conducted in the absence of any commercial or financial relationships that could be construed as a potential conflict of interest.
